# Metagenomic sequencing in intracranial pyogenic infections: limitations in viability and implications for antimicrobial decision-making

**DOI:** 10.1097/MS9.0000000000005586

**Published:** 2026-08-25

**Authors:** Sabeen Tahir, Zohaib Hassan, Ayesha Tariq, Rejina Chhetri, Janu Chhetri, Marina Kc

**Affiliations:** a Federal Medical and Dental College, Islamabad, Pakistan; b Punjab Medical College, Faisalabad, Punjab, Pakistan; c Nepalgunj Medical College, Kohalpur, Banke, Nepal; d Department of Medicine, Parbat District Hospital, Badagaoon Sadak, Kushma, Nepal

*To the Editor,*

The management of intracranial pyogenic infections remains a major challenge in neuroinfectious disease treatment due to their rapid clinical progression, risk of permanent neurological damage, and high mortality rates despite advances in medical therapy. Brain abscesses and similar suppurative intracranial infections continue to be associated with substantial morbidity and case-fatality rates approaching 10–20%. This highlights the necessity for prompt and accurate identification of causative pathogens. In this context, metagenomic next-generation sequencing (mNGS) has emerged as a promising diagnostic tool, capable of detecting a broad range of microorganisms directly from clinical samples, facilitating earlier and more accurate microbiological diagnosis of central nervous system (CNS) infections^[^1^]^.

The increasing use of mNGS in neuroinfectious diseases stems from its ability to identify a broad range of pathogens directly from clinical specimens without requiring prior assumptions regarding the causative agent^[^2^]^. Unlike conventional culture-based methods, mNGS is less affected by previous antimicrobial exposure and has shown improved detection of rare, fastidious, and polymicrobial CNS infections. Recent studies have demonstrated higher pathogen positivity rates with mNGS than standard microbiological techniques, underscoring its value in difficult cases^[^3^]^. Even with its diagnostic advantages, the inability of mNGS to determine microbial viability remains a significant and often overlooked challenge.

Despite its diagnostic strengths, mNGS possesses an important limitation: it identifies microbial nucleic acids but cannot reliably determine whether the detected organisms are viable and actively causing infection^[^2^]^. Residual DNA from nonviable pathogens may persist even after successful antimicrobial therapy, indicating that a positive mNGS result does not always reflect ongoing disease activity^[^4^]^. This distinction is particularly relevant in intracranial pyogenic infections, where treatment decisions often depend on accurate assessment of infection persistence.

The inability to accurately guide treatment duration on the basis of mNGS findings alone poses a substantial obstacle in clinical practice. Overreliance on molecular results may contribute to unnecessary prolongation or broadening of antimicrobial therapy, leading to adverse drug events, increased healthcare costs, and antimicrobial resistance^[^5^]^. These concerns highlight the importance of integrating mNGS findings into a comprehensive clinical framework rather than using them as a standalone determinant of therapeutic decisions.

To address these challenges, mNGS findings should be interpreted alongside clinical presentation, neuroimaging, inflammatory markers, and conventional microbiological investigations rather than in isolation. Additionally, emerging viability-sensitive approaches such as RNA-based sequencing and viability polymerase chain reaction may improve the distinction between active infection and residual microbial signatures, thereby refining antimicrobial management strategies.

In conclusion, although mNGS has transformed the diagnostic landscape of intracranial pyogenic infections, its inability to differentiate viable from non-viable microorganisms limits its role as a stand-alone tool for guiding antimicrobial therapy. Integrating molecular findings with clinical and conventional microbiological data will be essential to optimize patient management and fully harness the potential of this promising technology.

## Data Availability

No datasets were generated or analyzed during the preparation of this Letter to the Editor.

## Research registration unique identifying number

Not applicable for Letter to the Editor as per journal.
